# The acute effect and lag effect analysis between exposures to ambient air pollutants and spontaneous abortion: a case-crossover study in China, 2017–2019

**DOI:** 10.1007/s11356-022-20379-8

**Published:** 2022-05-06

**Authors:** Wenzheng Zhou, Xin Ming, Qing Chen, Xiaoli Liu, Ping Yin

**Affiliations:** 1 Chongqing Health Center for Women and Children, Chongqing, 401147 China; 2 Institute of Toxicology, College of Preventive Medicine, Army Medical University (Third Military Medical University), Chongqing, 400038 China; 3 School of Public Health, Tongji Medical College, Huazhong University of Science and Technology, Wuhan, 430030 China

**Keywords:** Air pollution, Exposure, Spontaneous abortion, Case-crossover

## Abstract

**Introduction:**

Recent studies demonstrated that living in areas with high ambient air pollution may have adverse effects on pregnancy outcomes, but few studies have investigated its association with spontaneous abortion. Further investigation is needed to explore the acute effect and lag effect of air pollutants exposure on spontaneous abortion.

**Objective:**

To investigate the acute effect and lag effect between exposure to ambient air pollutants and spontaneous abortion.

**Methods:**

Research data of spontaneous abortion were collected from the Chongqing Health Center for Women and Children (CQHCWC) in China. The daily ambient air pollution exposure measurements were estimated for each woman using inverse distance weighting from monitoring stations. A time-stratified, case-crossover design combined with distributed lag linear models was applied to assess the associations between spontaneous pregnancy loss and exposure to each of the air pollutants over lags 0–7 days, adjusted for temperature and relative humidity.

**Results:**

A total of 1399 women who experienced spontaneous pregnancy loss events from November 1, 2016, to September 30, 2019, were selected for this study. Maternal exposure to particulate matter 2.5 (PM_2.5_), particle matter 10 (PM_10_) nitrogen dioxide (NO_2_), and sulfur dioxide (SO_2_) exhibited a significant association with spontaneous abortion. For every 20 μg/m^3^ increase in PM_2.5_, PM_10_, NO_2_, and SO_2_, the RRs were 1.18 (95% *CI*: 1.06, 1.34), 1.12 (95% *CI*, 1.04–1.20), 1.15 (95% *CI*: 1.02, 1.30), and 1.92 (95% *CI*: 1.18, 3.11) on lag day 3, lag day 3, lag day 0, and lag day 3, respectively. In two-pollutant model combined with PM_2.5_ and PM_10_, a statistically significant increase in spontaneous abortion incidence of 18.0% (*RR* = 1.18, 95% *CI*: 1.06, 1.32) was found for a 20 μg/m^3^ increase in PM_2.5_ exposure, and 11.2% (*RR* = 1.11, 95% *CI*: 1.03, 1.20) for a 20 μg/m^3^ increase in PM_10_ exposure on lag day 3, similar to single-pollutant model analysis.

**Conclusion:**

Maternal exposure to high levels of PM_2.5_, PM_10_, NO_2_, and SO_2_ during pregnancy may increase the risk of spontaneous abortion for acute effects and lag effects. Further research to explore sensitive exposure time windows is needed.

## Introduction

Nowadays, many studies have reported that ambient air pollution exposure is related to adverse pregnancy outcomes (Chen et al. 2021; Guo et al. 2019a; Zhu et al. 2015), including preterm birth (Li et al. 2017), low birth weight, stillbirth (Bekkar et al. 2020), and abortion (Enkhmaa et al. 2014b). But few studies have investigated the association between maternal exposure to ambient air pollution and spontaneous abortion (Di Ciaula and Bilancia 2015b; Moridi et al. 2014). Spontaneous abortion, also called spontaneous pregnancy loss, was defined as a subsequent negative urine pregnancy test after a positive test, a clinically confirmed pregnancy loss, or onset of menstruation depending on gestational age (Dastoorpoor et al. 2018). Recent literature has demonstrated that air pollutants exposure increases the risk of spontaneous abortion, but the results vary by pollutants and demographic factors (Ha and Mendola 2019; Moridi et al. 2014). The sensitive time window of exposure remains inconsistent in different studies. Further research is still essential to explore the effect of air pollutants exposure on spontaneous abortion, especially the lag effect and acute effect.

There is a lot of literature related to air pollutants exposure and pregnancy outcomes in China (Fang et al. 2020; Guo et al. 2019b; Zhang et al. 2016), but the association between exposure to ambient air pollutants and spontaneous abortion is still rarely reported. Chongqing is the largest municipality in China, with a permanent population of 31 million, an industrial city located along the Yangtze River with 40 districts. Compared with other cities in China, Chongqing formerly has poor air quality, known as the famous “Fog City.” The air quality of Chongqing has been improving since 2014 through the gradually strengthened management by the local government. Chongqing’s air quality has greatly improved in 2019 according to the monitoring indicators.

The special air quality changes in Chongqing city provide a unique research environment different from European and American countries and also give us a peculiar sample to study the effect of air pollution exposure on spontaneous abortion.

Therefore, we conducted the study with a large sample size and precise individual exposure assessment to explore the risk of spontaneous abortion with exposure to air pollutants in a special Chinese city. It focused on the purpose to assess the lag effect and acute effect and to estimate the individual and combined effects of air pollutants.

## Materials and methods

### Study population

Research data about the cases of spontaneous abortion were collected from the Chongqing Health Center for Women and Children (CQHCWC), which is the biggest maternity referral hospital in Chongqing. It received hospitalized patients with spontaneous abortion across the whole of Chongqing city, providing us with large-scale and widely distributed cases for research. The cases of spontaneous abortion were identified in CQHCWC using the following diagnosis codes: ICD-10 — O03.300 × 061, O03.4-O03.9 (Leiser et al. 2019). All the research women were diagnosed as spontaneous abortion or incomplete spontaneous abortion, with or without complications. A total of 1794 spontaneous abortion cases in the medical record room of CQHCWC from 2017 to 2019 were collected. Women over the age of 35 years old are more likely to suffer pregnancy loss because old age is a severe risk factor for spontaneous abortion (DeVilbiss et al. 2020; Eroglu et al. 2020; Sileo et al.). To avoid interference, this study has eliminated them. The cases whose gestational age was unknown and the fertilization time cannot be estimated were excluded. The events that occurred to women residing outside Chongqing at fertilization time were excluded. For women who experienced two or more than two pregnancy abortion events, only the first event if the two occurred within 14 weeks was included. Because whether a second observation was truly another independent pregnancy loss event or related to the first event was unable to verify. Finally, the sample consisted of 1399 spontaneous abortion events that were selected for the final analysis after a series of exclusion criteria. This study was approved by the institutional ethical committee board of CHCWC.

### Air pollution exposure and other covariate data

Air pollution concentration data, including PM_2.5_, PM_10_, SO_2_, carbon monoxide (CO), NO_2_, and ozone (O_3_), were measured at the air monitoring stations for the period from January 1, 2016, to December 30, 2019, in 9 main districts of Chongqing, China. The air pollution data was all collected from the Chinese national urban air quality monitoring platform published by the China National Environmental Monitoring Station of the Ministry of Ecology and Environment (http://www.zhb.gov.cn). The data of O_3_ and CO was incomplete, so only PM_2.5_, PM_10_, NO_2_, and SO_2_ were analyzed in this study. The exposure time of acute-effect in this study was estimated at the fertilization time, which was calculated according to the time of the last menstruation of every woman (Leiser et al. 2019).

Based on the detailed residence address of every research woman and the location of air monitoring stations, we got each latitude and longitude through the Amap open platform (https://lbs.amap.com/). Then according to their exact spatial locations in the digital map, we calculated the distance between each woman’s residence and monitoring sites by using ArcGIS (version 10.2). Daily ambient air pollution exposure measurements were estimated for each woman by inverse distance weighting of all observations from monitoring stations (Leiser et al. 2019). The benefit was that we were able to assign exposure values at an individual level, rather than distinct-level measurements from the raw data.

The weather data was collected from the National Weather Data Sharing System (http://data.cma.cn/). Daily mean temperature (°C) and relative humidity (%) were used for analysis. All analyses were controlled for confounding factors, such as daily average temperature, age (years), previous pregnancy history (yes or no), and race/ethnicity. Daily exposure measurements were calculated as the average concentrations of the day of all the spontaneous abortion cases and the preceding lags 0–7 days of air pollutants (Leiser et al. 2019).

### Statistical analysis

A case-crossover study was designed for analyzing the association between short-term air pollution exposure and the risk of spontaneous pregnancy loss. This kind of design has been widely used in similar research fields, characterized by selecting a case or event date as her own control (Janes et al. 2005). Time-stratified referent selection was applied because it can control for the season, month, and day of the week. The same days of the other week in the same month for the event day of each individual were chosen as referent days. For example, if the conception day of an object was Monday, the referent days were another several Monday in the same month. Then there were three or four referent periods per event day. This design increased efficiency, avoided overlap bias and time-trend bias, and matched on time-dependent confounders.

Relative risks (RRs) and 95% confidence intervals (CIs) for a 20 μg/m^3^ increase in PM_2.5_, PM_10_, SO_2_, and NO_2_ exposures were calculated. Spearman’s correlation coefficients were used to evaluate the associations between each pair of air pollutants. The associations between air pollution and spontaneous abortion were estimated by using the generalized linear model and distributed lag linear model (DLM) (Ananth et al. 2018; Leiser et al. 2019). To take into account possible over-dispersion of daily spontaneous abortion counts, we used quasi-Poisson estimation. We used R version 3.4.2 with “dlnm” package (version 2.1.3) for data statistical analysis (Ananth et al. 2018). All statistical tests were two-sided. A probability value of < 0.05 was considered statistical significance.

## Result

### Descriptive statistics of women and ambient air pollutants

The 1399 research women in the study ranged in age from 18 to 35 years old, with an average age of 28 years old. Among them, 35.7% of women had not been pregnant before, 29.4% had a previous pregnancy, and 34.9% had two or more pregnancies. More than two thirds (72.1%) of women had never given birth before. The mean (standard deviation) abortion gestational age was 11.3 (3.5) weeks.

The descriptive statistics for ambient air pollutants PM_2.5_, PM_10_, SO_2_, NO_2_, average temperature, and relative humidity at lag day 0 for spontaneous abortion case days were displayed (Table 1). The mean concentration and their corresponding ranges (min–max) were 33.61 μg/m^3^ (7.96–177.91 μg/m^3^) for PM_2.5_, 65.76 μg/m^3^ (11.85–241.14 μg/m^3^) for PM_10_, 43.31 μg/m^3^ (15.08–89.81 μg/m^3^) for NO_2_, 9.32 μg/m^3^ (3.35–33.96 μg/m^3^) for SO_2_, 19.33 ℃ (4.50–36.50 ℃) for apparent mean temperature and 75.26% RH (37.00–97.00% RH) during the entire study period. All pollutant correlations were positive. Correlations between air pollutants and meteorological factors were mostly negative, except for PM_2.5_ and humidity. A positive correlation between PM_2.5_ and PM_10_ (*r* = 0.957), and a negative correlation between PM_2.5_ and average daily temperature (*r* =  − 0.493) were observed (Table 2).

**Table 1:** Descriptive statistics for ambient air pollutants

Pollutants	Mean	SD	Median	Minimum	Maximum	Range	Skew	Kurtosis
PM_2.5_ (μg/m^3^)	41.1	26.58	33.61	7.96	177.91	169.95	1.81	3.89
PM_10_ (μg/m^3^)	65.76	36.16	57.53	11.85	241.14	229.29	1.47	2.76
NO_2_ (μg/m^3^)	43.31	13.33	41.59	15.08	89.81	30.62	1.41	3.32
SO_2_ (μg/m^3^)	9.32	4.06	8.41	3.35	33.98	30.62	1.41	3.32
Temperature (℃)	19.33	7.91	19.10	4.50	36.50	32.00	0.16	− 1.06
Humidity (%RH)	75.26	11.44	77.00	37.00	97.00	60.00	− 0.58	− 0.19

Abbreviations: *SD*, standard deviation. Note: Average concentrations of pollutants from inverse distance weighting of all observations (1399 women) from monitoring stations, Chongqing, China, November 1, 2016 to September 30, 2019

**Table 2:** Spearman’s correlation coefficients between ambient air pollutants and weather conditions

Pollutants	PM_2.5_	PM_10_	NO_2_	SO_2_	Temperature	Humidity
PM_2.5_	1					
PM_10_	0.957^**^	1				
NO_2_	0.747^**^	0.807^**^	1			
SO_2_	0.633^**^	0.721^**^	0.690 ^**^	1		
Temperature	− 0.493^**^	− 0.358^**^	− 0.324^**^	− 0.211^**^	1	
Humidity	0.044	− 0.184^**^	− 0.067^*^	− 0.312^**^	− 0.418^**^	1

Spearman’s correlation coefficients among daily average concentrations of air pollutants and meteorological factors in the study in Chongqing, China, November 1, 2016 to September 30, 2019. **denotes *P* < 0.001; *denotes *P* < 0.05

### Concentration changes of ambient air pollutants for women

**Fig. 1:**
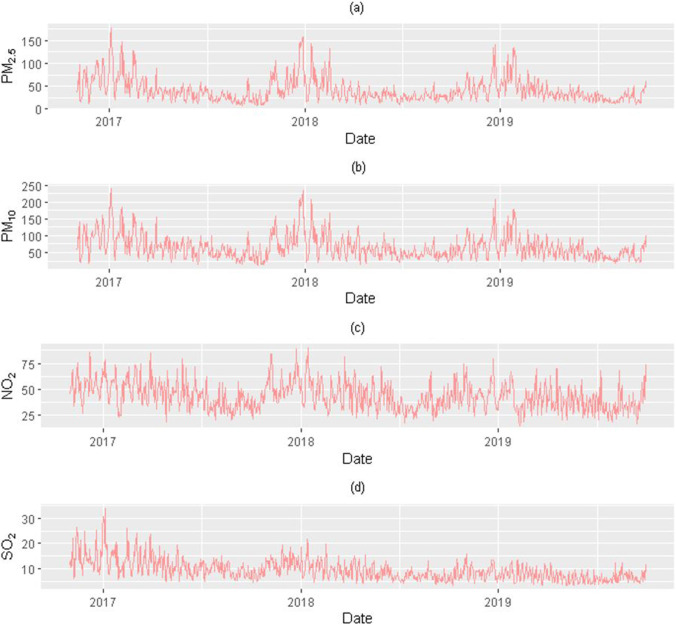
Daily averages of air pollutants in Chongqing, China, November 1, 2016 to September 30, 2019

Figure 1 shows the daily concentration variation trend of PM_2.5_, PM_10_, SO_2_, and NO_2_ of the 1399 women from 2016 to 2019 (the time of fertilization was calculated by the time of last menstruation, so the exposure time moved forward as a whole). The daily concentration of each ambient air pollutant was the mean value of the concentration of all spontaneous abortion cases on that day (calculated by the inverse distance weighted for each case). The results indicated that the seasonal trend is obvious, and the air quality is gradually improving.

### Associations between air pollutants exposure and spontaneous abortion

The relationship between the crude RRs for spontaneous pregnancy loss and the exposure of the gaseous air pollutants for lag effects and acute effects are shown in Table 3. For each 20 μg/m^3^ PM_2.5_ increase, the relative risk of spontaneous pregnancy loss increased by 18.4% (*RR* = 1.184, 95% *CI*: 1.06, 1.34) on lag day 3. For every 20 μg/m^3^ increase in PM_10_, NO_2_, and SO_2_, there were 11.5% (*RR* = 1.115, 95% *CI*: 1.04, 1.20), 14.9% (*RR* = 1.149, 95% *CI*: 1.02, 1.30), and 91.7% (*RR* = 1.917, 95% *CI*: 1.18, 3.11) increase in spontaneous abortion incidence on lag day 3, lag day 0, and lag day 3, respectively. The results of the adjusted model for apparent mean temperature and humidity were consistent with the previous crude model (Table 3).

**Table 3:** Crude and adjusted RRs and 95% CIs for spontaneous pregnancy loss and maternal air pollutants exposure

Pollutants	Lag	Crude *RR* (95% *CI*)	*p* value	Adjusted *RR* (95% *CI*)^a^	*p* value
PM_2.5_	lag0	1.182 (0.941, 1.483)	0.196	1.071 (0.986, 1.164)	0.160
	lag1	0.906 (0.809, 1.015)	0.145	0.906 (0.808, 1.015)	0.146
	lag2	1.004 (0.898, 1.121)	0.923	1.004 (0.898, 1.121)	0.923
	lag3	1.184 (1.060, 1.323)	0.034	1.183 (1.059, 1.322)	0.035
	lag4	0.849 (0.757, 0.951)	0.051	0.848 (0.757, 0.951)	0.050
	lag5	0.978 (0.876, 1.091)	0.638	0.976 (0.874, 1.090)	0.614
	lag6	1.084 (0.976, 1.205)	0.183	1.085 (0.976, 1.206)	0.180
	lag7	1.017 (0.941, 1.100)	0.619	1.015 (0.938, 1.099)	0.659
PM_10_	lag0	1.032 (0.977, 1.090)	0.279	1.033 (0.975, 1.093)	0.283
	lag1	0.954 (0.885, 1.027)	0.245	0.954 (0.885, 1.028)	0.247
	lag2	0.998 (0.928, 1.073)	0.941	0.998 (0.928, 1.073)	0.941
	lag3	1.115 (1.035, 1.200)	0.038	1.114 (1.035, 1.199)	0.038
	lag4	0.908 (0.842, 0.979)	0.058	0.907 (0.842, 0.978)	0.055
	lag5	0.971 (0.904, 1.044)	0.403	0.970 (0.903, 1.043)	0.391
	lag6	1.055 (0.984, 1.131)	0.181	1.055 (0.984, 1.131)	0.181
	lag7	1.017 (0.965, 1.072)	0.491	1.015 (0.963, 1.071)	0.537
NO_2_	lag0	1.149 (1.019, 1.295)	0.046	1.152 (1.019, 1.303)	0.048
	lag1	0.909 (0.784, 1.055)	0.240	0.912 (0.785, 1.059)	0.255
	lag2	0.976 (0.841, 1.133)	0.696	0.976 (0.841, 1.134)	0.697
	lag3	1.075 (0.926, 1.247)	0.340	1.073 (0.924, 1.245)	0.350
	lag4	0.947 (0.816, 1.100)	0.445	0.946 (0.814, 1.099)	0.440
	lag5	1.021 (0.879, 1.187)	0.735	1.019 (0.876, 1.186)	0.759
	lag6	0.933 (0.804, 1.083)	0.356	0.931 (0.802, 1.082)	0.346
	lag7	1.087 (0.963, 1.227)	0.217	1.084 (0.960, 1.224)	0.229
SO_2_	lag0	0.838 (0.547, 1.285)	0.399	0.822 (0.515, 1.311)	0.394
	lag1	1.039 (0.629, 1.717)	0.844	1.052 (0.637, 1.737)	0.799
	lag2	0.843 (0.511, 1.392)	0.469	0.844 (0.511, 1.392)	0.472
	lag3	1.917 (1.182, 3.111)	0.040	1.913 (1.179, 3.104)	0.041
	lag4	0.681 (0.412, 1.127)	0.183	0.674 (0.407, 1.116)	0.176
	lag5	0.659 (0.400, 1.085)	0.156	0.655 (0.398, 1.079)	0.152
	lag6	1.215 (0.754, 1.959)	0.404	1.209 (0.750, 1.949)	0.414
	lag7	0.979 (0.643, 1.491)	0.894	0.955 (0.626, 1.458)	0.785

RRs were estimates based on per 20 μg/m^3^ increase in PM_2.5_, PM_10_, NO_2_, and SO_2_. ^a^Adjusted for average temperature and relative humidity

### Results of combined effect analysis of two-pollutant models

The results of combined effect analysis for two-pollutant models are shown in Table 4. After adjusting apparent mean temperature and humidity, the association between two air pollutants and spontaneous abortion was calculated (Ananth et al. 2018). In a two-pollutant model combined with PM_2.5_ and PM_10_, a statistically significant increase in spontaneous abortion incidence of 18.0% (*RR* = 1.18, 95% *CI*: 1.06, 1.32) was found for a 20 μg/m^3^ increase in PM_2.5_ exposure, and 11.2% (*RR* = 1.112, 95% *CI*: 1.03, 1.20) for a 20 μg/m^3^ increase in PM_10_ exposure on lag day 3, similar to single-pollutant model analysis. As shown in Table 4, combined with PM_2.5_ and NO_2_, there were 18.7% (*RR* = 1.187, 95% *CI*: 1.06, 1.33) and 14.8% (*RR* = 1.148, 95% *CI*: 1.00, 1.31) significant increase in spontaneous abortion incidence on lag day 3 and lag day 0 respectively. Similarly, for PM_2.5_ and SO_2_, the RRs were 1.18 (95% *CI*: 1.06, 1.32) and 1.88 (95% *CI*: 1.16, 3.06) both on lag day 3 with statistical significance.

**Table 4:** Percent change in risk (and 95% *CI*) of incidence per 20 μg/m^3^ increase in two-pollutant models, adjusted for humidity and apparent mean temperature, Chongqing, China, 2017–2019: Distributed lag linear model

Pollutants	Lag	Adjusted RR (95%CI)	*p* value
PM_2.5_ + PM_10_			
PM_2.5_	lag0	1.182 (0.941, 1.483)	0.196
	lag1	0.902 (0.805, 1.011)	0.135
	lag2	1.004 (0.899, 1.122)	0.916
	lag3	1.180 (1.057, 1.318)	0.036
	lag4	0.849 (0.757, 0.951)	0.051
	lag5	0.977 (0.875, 1.090)	0.620
	lag6	1.084 (0.976, 1.205)	0.182
	lag7	1.014 (0.937, 1.098)	0.670
PM_10_	lag0	0.952 (0.817, 1.109)	0.487
	lag1	0.949 (0.880, 1.023)	0.213
	lag2	0.998 (0.928, 1.074)	0.945
	lag3	1.112 (1.032, 1.197)	0.042
	lag4	0.907 (0.842, 0.978)	0.056
	lag5	0.970 (0.902, 1.043)	0.392
	lag6	1.055 (0.984, 1.131)	0.183
	lag7	1.015 (0.963, 1.070)	0.534
PM_2.5_ + NO_2_			
PM_2.5_	lag0	1.032 (0.937, 1.137)	0.484
	lag1	0.912 (0.813, 1.022)	0.166
	lag2	1.003 (0.897, 1.120)	0.949
	lag3	1.187 (1.062, 1.326)	0.033
	lag4	0.851 (0.759, 0.954)	0.053
	lag5	0.977 (0.875, 1.091)	0.629
	lag6	1.085 (0.976, 1.206)	0.182
	lag7	1.018 (0.941, 1.102)	0.600
NO_2_	lag0	1.148 (1.004, 1.312)	0.012
	lag1	0.910 (0.781, 1.06)	0.043
	lag2	0.976 (0.840, 1.134)	0.696
	lag3	1.072 (0.923, 1.245)	0.356
	lag4	0.946 (0.814, 1.099)	0.438
	lag5	1.019 (0.876, 1.186)	0.753
	lag6	0.931 (0.801, 1.082)	0.347
	lag7	1.084 (0.960, 1.224)	0.229
PM_2.5_ + SO_2_			
PM_2.5_	lag0	1.115 (1.010, 1.230)	0.086
	lag1	0.901 (0.804, 1.010)	0.131
	lag2	0.998 (0.893, 1.115)	0.964
	lag3	1.182 (1.058, 1.320)	0.035
	lag4	0.847 (0.755, 0.949)	0.059
	lag5	0.973 (0.872, 1.087)	0.580
	lag6	1.082 (0.973, 1.202)	0.192
	lag7	1.017 (0.939, 1.100)	0.629
SO_2_	lag0	0.670 (0.393, 1.141)	0.188
	lag1	0.969 (0.581, 1.617)	0.872
	lag2	0.828 (0.501, 1.368)	0.434
	lag3	1.884 (1.159, 3.063)	0.045
	lag4	0.666 (0.401, 1.105)	0.168
	lag5	0.649 (0.393, 1.071)	0.147
	lag6	1.203 (0.745, 1.944)	0.425
	lag7	0.952 (0.622, 1.457)	0.774

RRs were estimates based on per 20 μg/m^3^ increase in PM_2.5_, PM_10_, NO_2_, and SO_2_. ^a^Distributed lag nonlinear models adjusted for average temperature and relative humidity

## Discussion

**Fig. 2:**
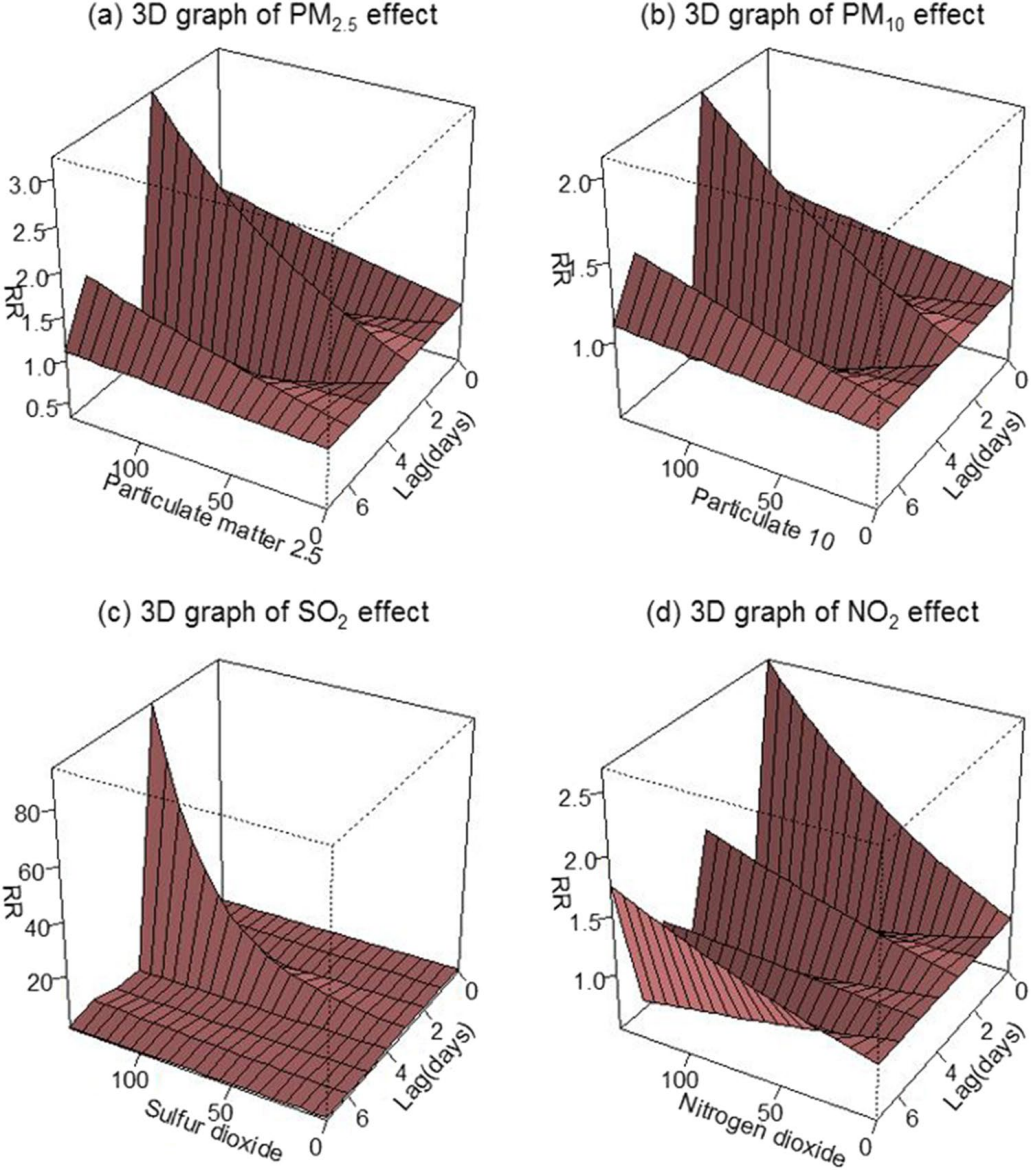
Rate ratio of spontaneous abortions associated with a 20-unit increase in air pollutant levels for different single-day lags of lag 0–7 days

This study carried out a time-stratified, case-crossover design with DLM model to evaluate the acute effect of air pollutants exposure on spontaneous abortion at each lag from 0 to 7 days of the calculated fertilization time. In particular, the city with special air quality changes was selected in this study with a larger sample size population. According to the results of this study, it was found that there were crucial associations between ambient air pollutants exposure and spontaneous pregnancy loss. Compared to the current literature, the present study is the largest sample size research on the relationship between ambient air pollutants and spontaneous abortion among the Chinese population. For every 20 μg/m^3^ increase in PM_2.5_, PM_10_, NO_2_, and SO_2_, the RRs were 1.18 (95% *CI*: 1.06, 1.34), 1.12 (95% *CI*, 1.04–1.20), 1.15 (95% *CI*: 1.02, 1.30), and 1.92 (95% *CI*: 1.18, 3.11) on lag day 3, lag day 3, lag day 0, and lag day 3, respectively (*p* < 0.05). The results of single-pollutant model and two-pollutant models adjusted for apparent mean temperature and humidity were similar. Results indicated an increase in the occurrence of spontaneous abortion associated with the exposure to ambient PM_2.5_, PM_10_, NO_2_, and SO_2_ because of their acute effect and lag effect (Figure 2).

The findings for PM_2.5_ and other air pollutants are generally consistent with those of existing related studies (Leiser et al. 2019; Liang et al. 2019; Zhang et al. 2019). In the first case-crossover study which investigates whether acute common air pollutants exposure trigger spontaneous pregnancy loss (Leiser et al. 2019), Leiser et al. found that a 10 μg/m^3^ increases in the average concentration of NO_2_ during a much shorter duration in time (3 days, 7 days) were associated with a 16% increase in the odds of spontaneous pregnancy loss. In a study conducted in China, Xue T and Zhang Q found that fertility rates were significantly decreased by 2.0% (95% *CI*: 1.8%, 2.1%) per 10 μg/m^3^ increment of PM_2.5_ (Xue and Zhang 2018). Another study reported a strong correlation between NO_2_ and spontaneous pregnancy loss (*r* > 0.8) (Enkhmaa et al. 2014a). In a systematic review by Conforti et al., they found elevated air pollution increased the risk of diminished fertility outcomes for the exposed population, including miscarriage and rates of live births (Conforti et al. 2018). A time-series study by Dastoorpoor et al. indicated a significant relationship between each 10 μg/m^3^ increase in SO_2_ and spontaneous abortion in lag 0 and 9 days using a quasi-Poisson distributed lag model (Dastoorpoor et al. 2018).

However, some inconsistencies between our study and previous research should be noticed (Ciaula and Bilancia 2015a; Frutos et al. 2015; Hou et al. 2014). In a study conducted by Moridi et al., findings demonstrated that odd ratios of abortion in the areas with higher concentrations of NO_2_ and PM_10_ were 0.96 and 1.01, respectively (*P* < 0.05); there was no significant association between prenatal exposure to SO_2_ and abortion (Moridi et al. 2014). Gaskins et al. conducted a case-crossover study with a sample size of 3585 women and found that an increase in PM_10_ (per 3.9 μg/m^3^) and PM_2.5_ (per 2.0 μg/m^3^) in the year before pregnancy was significantly associated with 1.12 (95% *CI*: 1.06, 1.19) and 1.10 (95% *CI*: 1.04, 1.17) higher odds of spontaneous abortion, respectively (Gaskins et al. 2019). The differences in the research methods and the component of air pollutants in different areas may cause various results (Raz et al. 2018; Wdowiak et al. 2018). This study with a large sample size and precise individual exposure assessment provided more convincing research evidence.

The focus of this study is not only on the associations between air pollutants level and spontaneous abortion with RRs but also on the combined effect of two-pollutant models. Due to the positive correlation between PM_2.5_ and PM_10_ (*r* = 0.96), NO_2_ (*r* = 0.75), and SO_2_ (*r* = 0.63), the results of two-pollutant models were similar to the univariate analysis. That is consistent with many articles that studied the relationship between ambient air pollutants and birth outcomes (Liu et al. 2019; Yang et al. 2020). The advantage value of case-crossover design is to control unobservable factors, i.e., the constant individual characteristics, such as maternal age at conception, living behaviors, season, and race (Ananth et al. 2018; Janes et al. 2005; Leiser et al. 2019). So the research results are reasonable and relatively reliable.

Additionally, some previous studies on air pollutants affecting adverse pregnancy outcomes, including spontaneous pregnancy loss, focused on exposure time in early pregnancy (Friedrich 2014; Hou et al. 2014; Patelarou and Kelly 2014). In this study, the short-term exposure windows before or at fertilization time were paid more attention, to attempt to assess the acute health effects of ambient air pollutants. Results indicated lag 3 day was the most statistically significant time for PM_2.5_, PM_10_, and SO_2_. In particular, the sensitive exposure time window for NO_2_ was lag 0 day both in crude and adjusted models.

The mechanism underlying the effect of air pollutants exposure on spontaneous abortion is still a matter of debate (Conforti et al. 2018; Jia and Guo 2014). Many studies reported the opinion that air pollutants had the effect of promoting oxidative stress and inflammatory processes (Grippo et al. 2018; Liang et al. 2020; Mahalingaiah 2018). Miscarriage may occur if there was an imbalance between active oxygen species and antioxidant defense system in embryonic tissues (Red et al. 2011). Some literature hypothesized that the result was caused by the mimic effect to androgens and estrogens of air pollutants in humans. The endocrine disruptors of air pollutants can act through classical nuclear receptors, but also through estrogen-related receptors, membrane-bound estrogen-receptors, and interaction with targets in the cytosol resulting in activation of the Src/Ras/Erk pathway and contributing to infertility (De Coster and van Larebeke 2012). In addition, some animal experimental studies have demonstrated that PM_2.5_ and PM_10_ exposure can increase oxidative stress (Blum et al. 2017; Li et al. 2013). The academic opinion about the genotoxic effect and DNA methylation influenced by air pollutants is also discussed a lot (Perin et al. 2010b). Therefore, according to the previous studies, our findings are biologically plausible.

This study has some limitations. First, we estimated ambient air pollutants exposure at residential addresses instead of actual personal exposure which may result in measurement error or bias. However, we have estimated the personal exposure value accurately by using inverse distance weighting for each woman and each day. Second, the lack of individual-level data in the medical record including socioeconomic condition, maternal education, and information on personal risk factors such as cigarette smoking, drinking, and diseases histories may lead to a potential bias in the results. Fortunately, a larger sample size was applied to adjust the bias to some extent. Third, this study only selected women cared in the CQHCWC. There were some women may have sought outpatient care in other hospitals. Additionally, some patients with early miscarriages may have been missed in medical records if the woman is unaware of the pregnancy (Moridi et al. 2014; Perin et al. 2010a). Furthermore, it only explored lag days 0–7 exposure time windows for estimating acute effect in this study. More sensitive exposure time windows during the whole pregnancy period should be further researched.

## Conclusion

In summary, this study demonstrated that maternal exposure to high levels of PM_2.5_, PM_10_, NO_2_, and SO_2_ may increase the risk of spontaneous abortion through special air quality changes, a large sample size, and precise individual exposure assessment. It may have acute harmful effects and lag effects on pregnancy outcomes a few days before fertilization. Further research to explore sensitive exposure time windows is needed. Should we propose ideas and schemes to reduce the adverse effects of ambient air pollutants on pregnancy outcomes.

## Data Availability

The datasets that support the findings of this study are openly available from the corresponding author on reasonable request.

## Abbreviations

PM_2.5_: Particulate matter 2.5
PM_10_: Particulate 10
SO_2_: Sulfur dioxide
NO_2_: Nitrogen dioxide
CO: Carbon monoxide
O_3_: Ozone
DLM: Distributed lag linear model
CQHCWC: Chongqing Health Center for Women and Children
RR: Relative risk
CI: Confidence interval
SD: Standard deviation
